# Clinical Features, Antibody Profiles, and Prognostic Factors in Autoimmune Encephalitis: A Single-Center Study

**DOI:** 10.3390/jcm14196806

**Published:** 2025-09-26

**Authors:** Bedriye Karaman, Gülcan Neşem Baskan, Merve Yavuz, Ayşe Güler, Özgül Ekmekci, Nur Yüceyar, Rasim Tunçel

**Affiliations:** 1 Neurology Department, Faculty of Medicine, Ege University, Izmir 35100, Turkey; bedriye.karaman@ege.edu.tr (B.K.); merve.yavuz@ege.edu.tr (M.Y.); ayse.guler@ege.edu.tr (A.G.); ozgul.ekmekci@ege.edu.tr (Ö.E.);; 2 Neurology Department, Izmir Bozyaka Training and Research Hospital, Izmir 35170, Turkey

**Keywords:** autoimmune encephalitis, limbic encephalitis, prognostic factors

## Abstract

**Background/Objectives**: Autoimmune encephalitis (AIE) comprises a heterogeneous group of inflammatory central nervous system (CNS) disorders characterized by variable clinical presentations and antibody profiles. This study aimed to identify poor prognostic factors in AIE by retrospectively evaluating patients diagnosed based on clinical, radiological, and serological findings. **Methods**: Forty-four patients diagnosed with AIE between 2014 and 2024 were included. Demographic, clinical, radiological, and serological data were collected retrospectively. Patients were grouped based on antibody localization (intracellular, surface, and seronegative) and classified by treatment response. Poor prognosis was defined as a lack of objective clinical improvement to treatment or death. **Results**: The mean age was 57.8 ± 13.6 years, with a female-to-male ratio of approximately 1:1. Limbic encephalitis (LE) was the most common clinical presentation (43.2%). Malignancy was detected in 33.3% of patients, most frequently in those with SOX1 (83.3%), anti-Hu (60.0%), and anti-Yo (50.0%) antibodies. Anti-SOX1 positivity was significantly associated with both malignancy (OR = 27.5, *p* = 0.007) and mortality (OR = 13.2, *p* = 0.009), while anti-LGI1 positivity correlated with the absence of malignancy (*p* = 0.036). Patients with LE showed significantly better treatment responses (OR = 14.0, *p* = 0.019). Mortality was 20.1% overall and highest among anti-SOX1-positive patients (66.7%). The presence of multiple antibodies was associated with higher mortality and poorer prognosis, although not statistically significantly. **Conclusions**: Anti-SOX1 positivity is a key indicator of poor prognosis in AIE and is strongly associated with both malignancy and mortality. In contrast, LE presentation was linked to a better treatment response. Antibody profile, clinical features, and malignancy screening are critical for risk stratification and guiding management in AIE.

## 1. Introduction

Autoimmune encephalitis (AIE) is an inflammatory condition resulting from the immune system attacking components of the central nervous system (CNS). The classification of AIE varies according to clinical findings, underlying antibodies, and overlapping encephalitis types [1]. AIE has been shown to have a prevalence comparable to infectious encephalitis, with an incidence of 0.2–0.8 per 100,000 person-years [2]. Although misdiagnosis persists, AIE is increasingly considered in the differential diagnosis of individuals exhibiting subacute cognitive decline, psychiatric symptoms, movement disorders, and seizures. The rise in AIE diagnosis is attributed to more widespread use of antibody testing and growing awareness among clinicians [3]. In recent years, with an increase in clinical studies and the publication of international guidelines, the likelihood of earlier diagnosis and treatment is improving [4]. Tumors and viral encephalitis are two potential triggers of AIE. Some involved cancers contain nerve tissue or express neuronal proteins targeted by autoantibodies, suggesting that ectopic expression may initiate the autoimmune response. Antibodies against the NMDAR and other neuronal cell surface proteins may cause recurrent neurologic symptoms weeks after herpes simplex encephalitis and possibly other types of viral encephalitis [5]. Cerebrospinal fluid (CSF) examination, brain magnetic resonance imaging (MRI), electroencephalography, and panel-based neural antibody testing are recommended for all patients suspected of having AIE [6].

In this study, we aimed to identify prognostic factors in AIE by retrospectively evaluating forty-four patients diagnosed with AIE based on clinical, serological, and radiological features.

## 2. Materials and Methods

Fifty patients who were investigated for possible AIE between 2014 and 2024 were included in this study. The patients’ demographic data, as well as their clinical, radiological, electrophysiological, and serological characteristics, were obtained retrospectively from electronic medical records. All patients underwent brain MRI, and the findings were classified as limbic pattern, striatal pattern, perivascular enhancement, diencephalic/brainstem involvement, cortical pattern, or MRI-negative, according to the involvement patterns described by Sanvito et al. [7]. Diagnostic criteria for possible AIE were described as the fulfillment of the following three criteria: (1) subacute (rapid progression of less than three months) onset of working memory deficits, altered mental status, or psychiatric symptoms; (2) new focal CNS findings, seizures not explained by a previously known seizure disorder, CSF pleocytosis (white blood cell count of more than five cells per mm^3^), or MRI features suggestive of encephalitis; (3) reasonable exclusion of alternative causes [8]. The antibody-negative but probable AIE in our cohort met all of the following criteria: (a) subacute working memory deficits, altered mental status, or psychiatric symptoms; (b) well-defined autoimmune encephalitis syndromes excluded; and (c) absence of well-characterized autoantibodies in serum and CSF, with at least two features among MRI abnormalities, CSF pleocytosis or oligoclonal bands/elevated IgG index, or inflammatory infiltrates in brain biopsy [9]. Six patients were excluded for failing to meet the diagnostic criteria.

The patients were classified into four groups according to their treatment response: death, no objective response, partial objective response (defined as improvement in less than 50% of clinical findings), and good clinical response (defined as improvement in more than 50% of clinical findings). Poor prognosis was defined as the absence of an objective clinical response to treatment or death.

The data were analyzed using SPSS version 25. Descriptive statistics and frequency distributions were calculated initially. For categorized data, the Chi-square test or Likelihood Ratio (LR) test was applied as appropriate. Non-parametric correlation analysis was performed, and based on the results, logistic regression analysis was conducted. For the group of 29 patients with intracellular antibodies, the same analyses—Chi-square or LR test, non-parametric correlation, and logistic regression modeling—were repeated. Statistical analysis could not be conducted separately for the extracellular antibody and seronegative groups due to insufficient sample sizes. *p*≤ 0.05 was considered statistically significant.

## 3. Results

The mean age of the 44 patients included in this study was 57.8 ± 13.60 years (range: 21–83), and 47.7% were female (*n* = 21). The average duration from symptom onset to the first medical consultation was 38.5 ± 66.83 weeks (range: 1–312). The time from the first medical consultation to a definitive diagnosis was found to be 8.7 ± 38.86 weeks (range: 1–260). The patients were divided into three groups based on the localization of the detected antibody: intracellular, surface, and seronegative. The groups’ descriptive are provided in Table 1.

Among the specific antibodies, anti-Yo (*n* = 10, 22.7%), anti-SOX1 (*n* = 6, 13.6%), anti-Hu (*n* = 5, 11.4%), anti-NMDAR (*n* = 5, 11.4%), anti-LGI1 (*n* = 5, 11.4%), anti-amphiphysin (*n* = 3, Among the specific antibodies, anti-Yo (*n* = 10, 22.7%), anti-SOX1 (*n* = 6, 13.6%), anti-Hu (*n* = 5, 11.4%), anti-NMDAR (*n* = 5, 11.4%), anti-LGI1 (*n* = 5, 11.4%), anti-amphiphysin (*n* = 3, 6.8%), anti-GAD (*n* = 3, 6.8%), and anti-titin (*n* = 3, 6.8%) were detected most frequently. No specific antibodies were identified in four patients. Other antibodies were detected in only one patient each. In our cohort, 6 patients (13.6%) had multiple antibody positivity. The most common co-occurring antibody was anti-titin, which was observed in 4.5% of patients.

Nineteen patients (43.2%) were diagnosed with limbic encephalitis (LE), eighteen (40.9%) with paraneoplastic cerebellar syndrome (PCS), three (6.8%) with paraneoplastic polyneuropathy (PP), two (4.5%) with opsoclonus–myoclonus syndrome (OMS), and two (4.5%) with stiff person syndrome (SPS) according to clinical presentation.

Radiological evaluation revealed that patients with a limbic pattern on MRI did not exhibit malignancy, and this finding was statistically significant (*p* = 0.036). Moreover, a correlation was observed between a limbic pattern on MRI and good treatment response (*p* = 0.030, r = 0.328; Spearman correlation). In contrast, no significant association was found between prognosis and normal MRI or other involvement patterns.

Malignancy screening was performed in 95.5% (*n* = 42) of the patients. Among these 42 patients, the malignancy rate was 33.3% (*n* = 14). No significant correlation was observed between gender and the presence of malignancy (r= −0.068, *p* = 0.67). According to clinical presentation, the frequency of malignancy was found to be 33.3% (*n* = 6) in patients with PCS, 27.8% (*n* = 5) in those with LE, 66.6% (*n* = 2) in patients with PP, and 50% (*n* = 1) in patients with OMS. No statistically significant difference was found between the groups. When evaluated according to antibody positivity, the frequency of malignancy was 83.3% (*n* = 5) in anti-SOX1-positive patients, 50.0% (*n* = 5) in anti-Yo positive patients, and 60.0% (*n* = 3) in anti-Hu-positive patients. The malignancy rate was 25.0% (*n* = 1) among seronegative patients. No malignancy was detected in patients positive for anti-LGI1, anti-GAD, or anti-amphiphysin antibodies. When compared with other patients, the absence of malignancy in anti-LGI1-positive cases was statistically significant (LR, *p* = 0.036).

Nine deaths were recorded among the forty-four participants, corresponding to a mortality rate of 20.1%. There was a significant correlation between presence of malignancy and mortality in patients with AEI (r = 0.492, *p* = 0.001). The highest mortality was observed in patients with anti-SOX1 antibodies (66.7%), followed by anti-Hu (40%), anti-amphiphysin (33%), and both anti-Yo and anti-NMDAR (20%) antibodies. Of the nine deaths, four occurred in anti-SOX1-positive patients, two in anti-Yo-positive patients, two in anti-Hu-positive patients, one in an anti-amphiphysin-positive patient, and one in an anti-NMDAR-positive patient. Patients with multiple specific antibody positivity (*n* = 6) had a higher mortality rate (50.0%) compared to those with a single antibody positivity (15.8%, *n* = 38), but this difference was not statistically significant (*p* = 0.077). However, anti-SOX1 positivity was significantly associated with higher mortality (*p* = 0.007). No deaths occurred in patients with anti-LGI1 or anti-GAD antibodies or in seronegative individuals, although these findings were not statistically significant when compared to the rest of the cohort (*p* = 0.118, *p* = 0.232, and *p* = 0.165, respectively). By clinical diagnosis, the mortality rate was 33.3% in PP, 22.2% in PCS, and 21.1% in LE patients, with no significant differences among the groups.

When treatment responses were evaluated according to antibody positivity, patients with multiple antibody positivity and those positive for anti-SOX1 had a significantly poorer prognosis (*p* = 0.014 and *p* = 0.003, respectively). No significant associations were found with other antibody positivity. A summary of all prognostic factors were provided in Table 2.

A logistic regression analysis of the entire patient cohort (*n* = 44) evaluated malignancy, good clinical response to treatment, and mortality (Table 3). On average, mortality was found to be 13.2 times higher in patients positive for SOX1 compared to those without SOX1 positivity (B = 2.58, *p* = 0.009, OR = 13.2, 95% CI = 1.89–91.90). Good clinical response to treatment was observed to be 14 times higher in patients diagnosed with LE compared to those without (B = 2.64, *p* = 0.019, OR = 14.0, 95% CI = 1.54–127.23), and the presence of malignancy was, on average, 27.5 times higher in patients positive for anti-SOX1 antibodies (B = 3.31, *p* = 0.007, OR = 27.5, 95% CI = 2.50–302.17) and 5.5 times higher in those positive for anti-Yo antibodies (B = 1.71, *p* = 0.041, OR = 5.5, 95% CI = 1.07–28.20).

Among patients with antibodies targeting intracellular proteins (*n* = 29), mortality was found to be an average of 9.5 times higher in those positive for anti-SOX1 compared to those without (B = 2.25, *p* = 0.028, OR = 9.5, 95% CI = 1.27–70.96). Similarly, the presence of malignancy was an average of 10.7 times higher in anti-SOX1-positive patients compared to those who were negative (B = 2.37, *p* = 0.046, OR = 10.71, 95% CI = 1.05–109.78).

## 4. Discussion

This retrospective study examined the clinical, radiological, and serological characteristics of 44 individuals with AIE and discovered significant predictors of poor prognosis. The primary findings indicate a notable correlation between anti-SOX1 antibody positivity and elevated mortality and malignancy, implying its potential function as a robust predictor of poor prognosis. On the other hand, LE correlated with improved treatment response and a reduced probability of underlying malignancy, suggesting a potentially more favorable disease course. Furthermore, patients with multiple antibody positivity displayed a poorer prognosis than those with a singular antibody. The study’s strengths include antibody subtype classification, logistic regression analysis of independent risk factors, and radiological and clinical correlations to better understand illness outcomes. These findings may inform clinical decision-making by revealing AIE predicting factors. Forty-four patients were included with a mean age of 57.8 ± 13.6 years and a female-to-male ratio of approximately 1:1. Patients with AIE show distinct patterns in age and sex distribution, which vary by antibody subtype. For example, anti-NMDAR-positive AIE is more common in the pediatric population or young adult females, whereas anti-LGI1-positive cases are more frequently observed in older males [10,11]. The most frequent antibodies in AIE detected were anti-NMDA-R, anti-GAD65, anti-LGI, and anti-MOG in previous studies. The frequency of these antibodies varies by population, age, and clinical context, but anti-NMDAR antibodies are consistently the most common across studies. [10]. In our cohort, the most frequently detected specific antibodies were anti-Yo (*n* = 10, 22.7%), followed by anti-SOX1 (*n* = 6, 13.6%), anti-Hu (*n* = 5, 11.4%), anti-NMDAR (*n* = 5, 11.4%), anti-LGI1 (*n* = 5, 11.4%), anti-amphiphysin (*n* = 3, 6.8%), anti-GAD (*n* = 3, 6.8%), and anti-titin (*n* = 3, 6.8%). These findings may be attributed to the fact that the study was conducted at a single center and involved a limited number of patients. In our cohort, 6 patients (13.6%) had multiple antibody positivity. In a multicenter cohort from China that included 189 patients diagnosed with antibody-positive AIE, the frequency of co-occurrence of two or more antibodies was reported to be 4.2% [12]. In generally, the frequency of multiple antibody positivity in AIE is low (3–7%) [13], but certain combinations, such as NMDAR+MOG in children, may be more common [14].

Many patients experience significant delays between symptom onset and medical evaluation or diagnosis, often due to the psychiatric or non-specific nature of early symptoms. In our cohort, the mean time from symptom onset to first medical consultation was 38.5 weeks, and the mean time from consultation to diagnosis was 8.7 weeks. In a study evaluating the duration from symptom onset to antibody testing in patients with AIE, the interval was reported to be approximately 67 weeks in patients diagnosed from 2007 to 2012, whereas it decreased to 10.5 weeks in those diagnosed between 2013 and 2016 [15]. Increased awareness and use of antibody testing have reduced diagnostic delays in recent years.

In our cohort, poor prognosis was not significantly associated with demographic factors such as age or gender, nor with clinical variables including malignancy status, symptom duration prior to admission, or time from admission to diagnosis. This contrasts with prior studies suggesting that older age may independently predict worse outcomes in both antibody-positive and seronegative AIE [16,17,18]. Similarly, although most studies found no gender effect, male sex has been identified as a potential risk factor in anti-NMDAR encephalitis [19,20]. Although symptom duration before treatment is not a consistent predictor of prognosis, delays in initiating immunotherapy are strongly associated with poorer outcomes [21]. Similarly, the interval from hospital admission to diagnosis does not independently predict prognosis, yet treatment delays—often related to diagnostic challenges—remain a key determinant of unfavorable outcomes [22]. These findings emphasize that early recognition and timely immunotherapeutic intervention are critical for improving prognosis, rather than focusing solely on demographic or baseline clinical characteristics.

Several previous studies have demonstrated that the presence of malignancy is associated with increased mortality in AIE. Consistently, in our cohort, a significant correlation was observed between the presence of malignancy and mortality, supporting the impact of malignancy on disease outcome. Malignancy was detected in 33.3% of patients in our cohort. The rate of malignancy varies depending on the specific antibody involved, diagnostic methods, and patient demographics, but overall, malignancy is reported in 6–25% of cases of AIE [23,24]. In our study, the highest malignancy rates were observed in patients with anti-SOX1 (83.3%), anti-Hu (60.0%), and anti-Yo (50.0%) antibodies. Multiple case reports and studies show that the presence of SOX1 antibodies in AIEis a strong predictor of underlying malignancy, especially small cell lung cancer (SCLC) [25,26,27]. Anti-Hu and anti-Yo antibodies are onconeural antibodies known to be associated with significant malignancy risk [28,29]. Logistic regression analyses showed that anti-SOX1 positivity increased the odds of malignancy by 27.5 times (*p* = 0.007) and anti-Yo positivity increased the odds of malignancy by 5.5 times (*p* = 0.041) in all cohorts. In the group of intracellular antibody-positive patients, the odds of malignancy were 10.7 times higher in anti-SOX1-positive patients (*p* = 0.046). There was a statistically significant association between anti-LGI1 positivity and the absence of malignancy, suggesting that patients with anti-LGI1 encephalitis were less likely to have an underlying cancer (*p* = 0.036). This finding aligns with previous studies, which report an association with malignancy in less than 10% of anti-LGI1 encephalitis cases [30,31]. In our cohort, nine patients died during follow-up, corresponding to a mortality rate of 20.1%. Overall, the mortality rate for AIE is reported to range from 6% to 19%, but it can be much higher for certain subtypes [32]. Mortality was the highest among anti-SOX1-positive patients (66.7%), followed by those with anti-Hu (40.0%) and anti-amphiphysin (33.0%) antibodies. In contrast, no deaths were observed in patients with anti-LGI1 or anti-GAD antibodies or in seronegative individuals. In previous studies, mortality was reported to be the highest for anti-GABABR-positive AIE, whereas it was the lowest in cases associated with anti-NMDAR and anti-LGI1 antibodies [33,34]. Mortality in AIE is influenced not only by the type of antibody but also by several other factors, including age at onset, clinical presentation, the presence of malignancy, and early treatment [34,35]. Anti-SOX1 positivity was significantly associated with increased mortality in this study. In a univariate analysis, the association between anti-SOX1 positivity and mortality was statistically significant (*p* = 0.007). Logistic regression analyses further supported this relationship: anti-SOX1 positivity increased the odds of mortality by 13.2 times in the entire cohort (*p* = 0.009) and by 9.5 times among patients with intracellular antibody positivity (*p* = 0.028). These findings suggest that the anti-SOX1 antibody may serve as an important predictor of poor prognosis in AIE, potentially due to its strong association with underlying malignancy. The high mortality observed in this subgroup aligns with recent publications indicating that intracellular (onconeural) antibodies—such as anti-Hu and anti-Yo—are frequently linked to cancer and associated with poor prognosis [36]. Patients with multiple antibody positivity had higher mortality (50.0%) compared to those positive for a single antibody (15.8%), though the difference was not statistically significant in our cohort (*p* = 0.077). Another recent study of 83 cases of AIE with multiple antibodies revealed a mortality rate of 31.3%, considerably higher than single-antibody AIE. The study determined that the presence of multiple antibodies substantially influences clinical characteristics, disease advancement, and prognosis, resulting in a considerable rise in mortality relative to instances with a single antibody [37]. Our results did not reach statistical significance, which may be attributed to the limited sample size. Treatment response analysis showed that patients with anti-SOX1 antibody or multiple antibody positivity had a significantly poorer prognosis (*p* = 0.003 and *p* = 0.014, respectively). The presence of multiple autoantibodies is associated with more complex disease and may increase the risk of poor outcomes, but large-scale outcome data are limited [14].

In this study, limbic involvement on MRI was significantly associated with the absence of malignancy, suggesting a predominance of non-paraneoplastic AIE in these cases. Additionally, limbic patterns were positively correlated with a better treatment response. In the logistic regression analysis, patients with LE were 14 times more likely to show a good clinical response to treatment (*p* = 0.019). This finding supports previous reports suggesting that AIE with predominant limbic involvement often responds favorably to immunotherapy. Limbic involvement may reflect a pathophysiological mechanism more amenable to immunomodulation compared to other encephalitic subtypes or paraneoplastic features [8].

This study has some limitations. Six patients who were seronegative for antibodies in the serum were excluded due to the absence of CSF analysis or lack of antibody testing in the CSF, which prevented confirmation of the diagnostic criteria. Consequently, the sample size was reduced, and groups such as seronegative and surface antibody-positive patients were too small for independent statistical analysis. The study primarily assessed treatment response and mortality, but long-term functional outcomes (e.g., cognitive function, quality of life, relapse rates) were not evaluated because of the retrospective design.

## 5. Conclusions

This study highlights the prognostic value of antibody profile and clinical features in AIE. Anti-SOX1 positivity was strongly associated with both malignancy and mortality, while patients with LE showed better treatment responses. Early diagnosis and antibody-based risk stratification may guide clinical management and improve outcomes.

## Figures and Tables

**Table 1:** Descriptive data of the entire cohort and each group according to antibody localization.

	Total (N = 44)		İntracellular Ab (N = 29)		Surface Ab (N = 11)		Seronegative (N = 4)		K-W
	Mean/Median	±SD/IQR	Mean/Median	±SD/IQR	Mean/Median	±SD/IQR	Mean/Median	±SD/IQR	*p*
Onset Age	57.82	±13.60	60.14	±9.60	49.36	±20.64	64.25	±1.50	0.27
Symptom Onset to Diagnosis in weeks	8.00	16.00	8.00	52.00	4.00	23.00	7.00	40.00	0.62
CSF Cell	4.00	5.00	4.00	4.00	15.00	19.00	1.50	7.00	0.40
CSF Protein	45.81	±23.28	41.90	±17.63	55.86	±34.91	48.75	±27.17	0.96
	N	%	N	%	N	%	N	%	X^2^
Female	21	47.7	14	48.3	5	45.5	2	50	0.983
Malignancy	14	31.8	12	41.4	1	9.1	1	25	0.156
Mortality	9	20.5	8	27.6	1	9.1	0	0	0.246
Good Response to Treatment	8	18.2	3	10.3	4	36.4	1	25	0.152
LE	19	43.2	7	24.1	10	90.9	2	50	0.001
PSS	18	40.9	17	58.6	1	9.1	1	25	0.003
PP	3	6.8	3	10.3					0.435
OMS	2	4.5					1	25	0.056
SPS	2	4.5	2	6.9					0.582

Ab: antibody; K-W: Kruskal–Wallis; X^2^: Chi-square; SD: standard deviation; IQR: interquartile range; CSF: cerebrospinal fluid; LE: limbic encephalitis; PSS: paraneoplastic cerebellar syndrome; PP: paraneoplastic polyneuropathy; OMS: opsoclonus–myoclonus syndrome; SPS: stiff person syndrome. No significant association was found between poor prognosis and age (r = −0.024, *p* = 0.878) or gender (r = −0.097, *p* = 0.533). Likewise, malignancy did not demostrate a relationship with poor prognosis (r = 0.181, *p* = 0.252). In addition, the duration of symptoms prior to admission showed no significant correlation with poor prognosis (r = 0.162, *p* = 0.293), and the interval from admission to diagnosis was not associated with poor prognosis (r = 0.073, *p* = 0.639). Taken together, these findings suggest that demographic factors such as age and gender, as well as clinical variables including malignancy status, symptom duration, and diagnostic delay, do not appear to exert a significant influence on prognosis in this patient cohort.

**Table 2:** Univariate analysis of prognostic risk factors.

Antibody		Mortality		*p*	Treatment Response		*p*	Malignancy		*p*
		No	Yes		Poor	Good		Absent	Present	
Anti-Titin	Negative	33 (80.5%)	8 (19.5%)	0.588	34 (82.9%)	7 (17.1%)	0.513	26 (66.7%)	13(33.3%)	1.000
	Positive	2 (66.7%)	1 (33.3%)		2 (66.7%)	1 (33.3%)		2 (66.7%)	1 (33.3%)	
Anti-SOX1	Negative	33 (86.8%)	5 (13.2%)	0.007	30 (78.9%)	8 (21.1%)	0.106	27 (75.0%)	9 (25.0%)	0.006
	Positive	2 (33.3%)	4 (66.7%)		6 (100%)	0		1 (16.7%)	5 (83.3%)	
Anti-Hu	Negative	32 (82.1%)	7 (17.9%)	0.284	32 (82.1%)	7 (17.9%)	0.912	26 (70.3%)	11(29.7%)	0.192
	Positive	3 (60.0%)	2 (40.0%)		4 (80.0%)	1 (20.0%)		2 (40.0%)	3 (60.0%)	
Anti-Yo	Negative	27 (79.4%)	7 (20.6%)	0.968	27 (79.4%)	7 (20.6%)	0.421	23 (71.9%)	9 (28.1%)	0.209
	Positive	8 (80.0%)	2 (20.0%)		9 (90.0%)	1 (10.0%)		5 (50.0%)	5 (50.0%)	
Anti-NMDAR	Negative	31 (77.5%)	9 (22.5%)	0.165	33 (84.6%)	6 (15.4%)	0.220	25 (64.1%)	14(35.9%)	0.111
	Positive	4 (100%)	0		3 (60.0%)	2 (40.0%)		3 (75.0%)	0	
Anti-LGI1	Negative	30 (76.9%)	9 (23.2%)	0.118	33 (84.6%)	6 (15.4%)	0.220	23 (62.2%)	14(37.8%)	0.036
	Positive	5 (100)	0		3 (60.0%)	2 (40.0%)		5 (100%)	0	
Seronegative	Negative	31 (77.5%)	9 (22.5%)	0.165	33 (82.5%)	7 (17.5%)	0.721	25 (65.8%)	13(34.2%)	0.704
	Positive	4 (100%)	0		3 (75.0%)	1 (25.0%)		3 (75.0%)	1 (25.0%)	
Anti-amphiphysin	Negative	33 (80.5%)	8 (19.5%)	0.588	34 (82.9%)	7 (17.1%)	0.513	25 (64.1%)	14(35.9%)	0.111
	Positive	2 (66.7%)	1 (33.3%)		2 (66.7%)	1 (33.3%)		3 (100%)	0	
Anti-GAD	Negative	32 (78.0%)	9 (22.0%)	0.232	33 (80.5%)	8 (19.5%)	0.263	26 (65.0%)	14(35.0%)	0.196
	Positive	3 (100%)	0		3 (100%)	0		2 (100%)	0	
Multipl antibody	Negative	32 (84.2%)	6 (15.8%)	0.077	31 (81.6%)	7 (18.4%)	0.917	25 (69.4%)	11(30.6%)	0.361
	Positive	3 (50%)	3 (50%)		5 (83.3%)	1 (16.7%)		3 (50%)	3 (50%)	
**Clinical Presentation**										
LE	Absent	20 (80%)	5 (20%)	0.932	24 (96.0%)	1 (4.0%)	0.004	15 (62.5%)	9 (37.5%)	0.506
	Present	15 (78.9%)	4 (21.1%)		12 (63.2%)	7 (36.8%)		13 (72.2%)	5 (27.8%)	
PSS	Absent	21 (80.8%)	5 (19.2%)	0.809	19 (73.1%)	7 (26.9%)	0.054	16 (66.7%)	8 (33.3%)	1.000
	Present	14 (77.8%)	4 (22.2%)		17 (94.4%)	1 (5.6%)		12 (66.7%)	6 (33.3%)	
PP	Absent	33 (80.5%)	8 (19.5%)	0.588	33 (80.5%)	8 (19.5%)	0.263	27 (69.2%)	12(30.2%)	0.220
	Present	2 (66.7%)	1 (33.3%)		3 (100%)	0		1 (33.3%)	2 (66.7%)	
OMS	Absent	33 (78.6%)	9 (21.4%)	0.332	34 (81.0%)	8 (19.0%)	0.364	27 (67.5%)	13(32.5%)	0.618
	Present	2 (100%)	0		2 (100%)	0		1 (50.0%)	1 (50.0%)	
SPS	Absent	27 (65.9%)	14(34.1%)	0.332	34 (81.0%)	8 (19.0%)	0.364	27 (65.9%)	14(34.1%)	0.364
	Present	1 (100%)	0		2 (100%)	0		1 (100%)	0	

LE: limbic encephalitis; PSS: paraneoplastic cerebellar syndrome; PP: paraneoplastic polyneuropathy; OMS: opsoclonus–myoclonus syndrome; SPS: stiff person syndrome.

**Table 3:** Logistic regression models.

		B	S.E.	*p*	Exp (B)	95% C.I. for EXP (B)	
**All cases**	Model for Mortality					Lower	Upper
	SOX1	2.58	0.99	0.009	13.2	1.896	91.907
	Constant	−1.887	0.48	0	0.152		
	Model for Good Response to treatment						
	LE	2.639	1.126	0.019	14	1.541	127.225
	Constant	−3.178	1.021	0.002	0.042		
	Model for Malignancy						
	SOX1	3.314	1.223	0.007	27.5	2.503	302.174
	Yo	1.705	0.834	0.041	5.5	1.073	28.198
	Constant	−1.705	0.544	0.002	0.182		
**İntracellular Ab-positive cases**	Model for Mortality						
	SOX1	2.251	1.026	0.028	9.5	1.272	70.964
	Constant	−1.558	0.55	0.005	0.211		
	Model for Malignancy						
	SOX1	2.372	1.187	0.046	10.714	1.046	109.784
	Constant	−0.762	0.458	0.096	0.467		

S.E.: standard error; C.I: confidence interval; LE: limbic encephalitis.

## Data Availability

The anonymized data can be accessed upon request to the corresponding author.

## Abbreviations

The following abbreviations are used in this manuscript:

AIE	Autoimmune encephalitis
CNS	Central nervous system
CSF	Cerebrospinal fluid
MRI	Magnetic resonance imaging
LE	Limbic encephalitis
PCS	Paraneoplastic cerebellar syndrome
PP	Paraneoplastic polyneuropathy
OMS	Opsoclonus–myoclonus syndrome
SPS	Stiff person syndrome
